# A dicentric chromosome identification method based on clustering and watershed algorithm

**DOI:** 10.1038/s41598-019-38614-7

**Published:** 2019-02-19

**Authors:** Xiang Shen, Yafeng Qi, Tengfei Ma, Zhenggan Zhou

**Affiliations:** School of Mechanical Engineering and Automation, Beihang University, Beijing, 100083 China

## Abstract

Aiming at the problem of low efficiency of dicentric chromosome identification counting under the microscope, this paper presents a joint processing algorithm combining clustering and watershed. The method first uses clustering and watershed algorithm to segment the original chromosome image, and then identifies the individual chromosomes. The results show that when the equivalent width Y parameter is selected m = 1, n = 1, the true positive rate of dicentric chromosome identification is 76.6%, and positive predictive value is 76.6% in high dose, which is higher than the threshold algorithm for the true positive rate (63.9%) and positive predictive value (63.5%). The number of identified dicentric chromosomes can be used for dose estimation. When 500 cells are used for identification and dose estimation, the dose estimation pass rate can reach 80% in high dose. But for low dose, more cells should be used to identify to increase the dose estimation pass rate.

## Introduction

**Figure 1:**
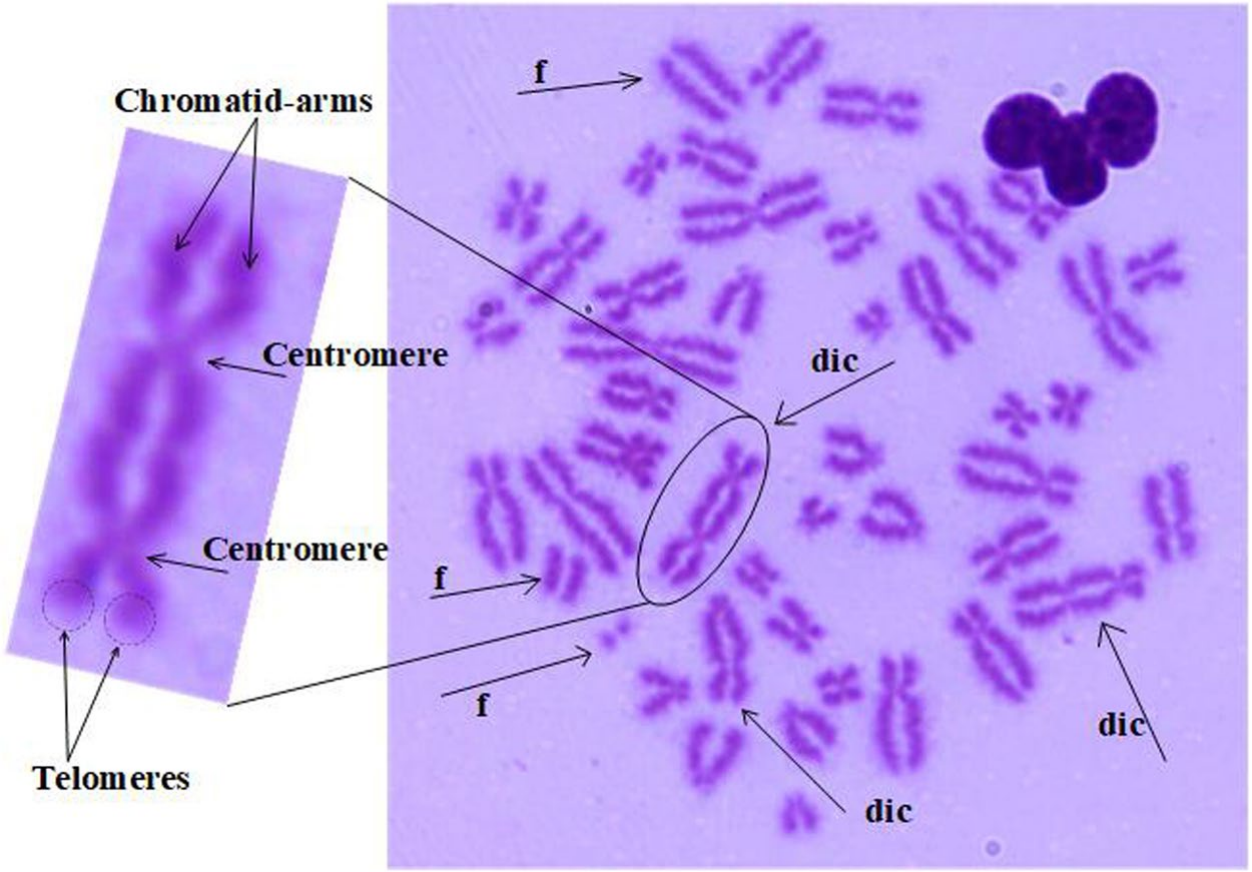
The structure of dicentric chromosome. Dic: dicentric chromosome. F: fragment.

Dicentric chromosome (dic) is the main type of aberration used for radiation dose estimation. Based on the number of dicentric chromosomes, it is possible to estimate the dose of radiation to an individual and thus to assess the work of regular radiation examinations. The health status of personnel, or when a radiation accident occurs, a treatment plan is formulated according to the radiation dose to save the lives of the radiation-affected personnel. The dicentric aberration is formed by the two linked broken chromosomes containing the centromeric parts, and also accompanied by an amphoteric body formed by the connection of the remaining chromosomes of the two chromosomes without a centromere fragment (f). For example, three dicentric chromosomes and three fragments are marked (Fig. 1). Dicentric chromosome identification is divided into two main steps: segmentation and identification. In these two steps, it is particularly important to segment a single chromosome from a chromosome clump. The quality of the segmentation result will influence the identification of the centromere point. Although many methods have been tried for chromosome segmentation, for example: an initial threshold for initial segmentation and then secondary segmentation based on the path density^1^; an improved classical fuzzy mean algorithm based on gain fields^2^; a cutting method based on geometry^3^; a method of using a white point approach^4^, the segmentation methods are not universally applicable due to many interference factors, and sometimes the segmented objects are also different. For example, there are image segmentation methods for M-FISH chromosome images^5,6^, but in this paper, the Giemsa staining chromosome images are used. The main steps to identify the centromeres are as follows: First, the central axis of a single chromosome is extracted, and then the centromeres are identified according to the characteristic parameters of the chromosome. Most of the centerline extraction methods are based on MAT (medial axis transformation) and different thinning methods^7–11^, such as distance transformation^7^, boundary extraction and refinement^8^. For the identification of centromeres, most of the methods are based on the characteristics of the centromeres, for example: geometric features^8^, the pixels of chromosomes^12–14^. Most of the current literature on chromosome segmentation is mainly for karyotype analysis, and there are a few papers for identifying and counting dicentric chromosomes, the DCScore software is used to identify the dicentric chromosomes and estimate radiation dose on a large accidental overexposure at Dakar, but the software has a 50% misrecognition rate for overlapping chromosomes^15^. The machine learning method is used to identify dicentric chromosomes, resulting in 50~65% true positive rate (TPR) and 70~80% positive predictive value (PPV)^16^. It is necessary to research and develop a method for improving the identification rate and accuracy.

When chromosomes are segmented, chromosomes will be a substantial loss for its shape and pixel linear compression by conventional methods such as threshold algorithm. Therefore, this paper uses the K-Means ++ clustering algorithm and the watershed algorithm to segment the chromosomes in cells. The clustering algorithm can remove the impurities in the original image and perform the initial segmentation. The watershed algorithm can further segment the lightly contiguous chromosomes so that the single chromosomes in the chromosome cluster can be completely segmented. Using this method can not only maintain the original morphology of chromosomes, but also effectively avoid the large number of pixel loss caused by linear compression in the segmentation process. And segmentation of a single chromosome or clumps only accounts for tens of KB, greatly reducing the generated process memory and improving the processing speed. The segmented single chromosomes are identified by centromeres. The algorithm has a true positive rate (TPR) 76.6% and a positive predictive value (PPV) of 76.6% in high dose. The number of identified dicentric chromosomes can be used to estimate the dose of the population exposed to the radiation source, for low dose radiation, more identified cells should be used, and for high dose radiation, the number of identified cells can be appropriately reduced. When 500 cells are used for identification and dose estimation, the dose estimation pass rate can reach 80% in high dose.

## Methods

**Figure 2:**
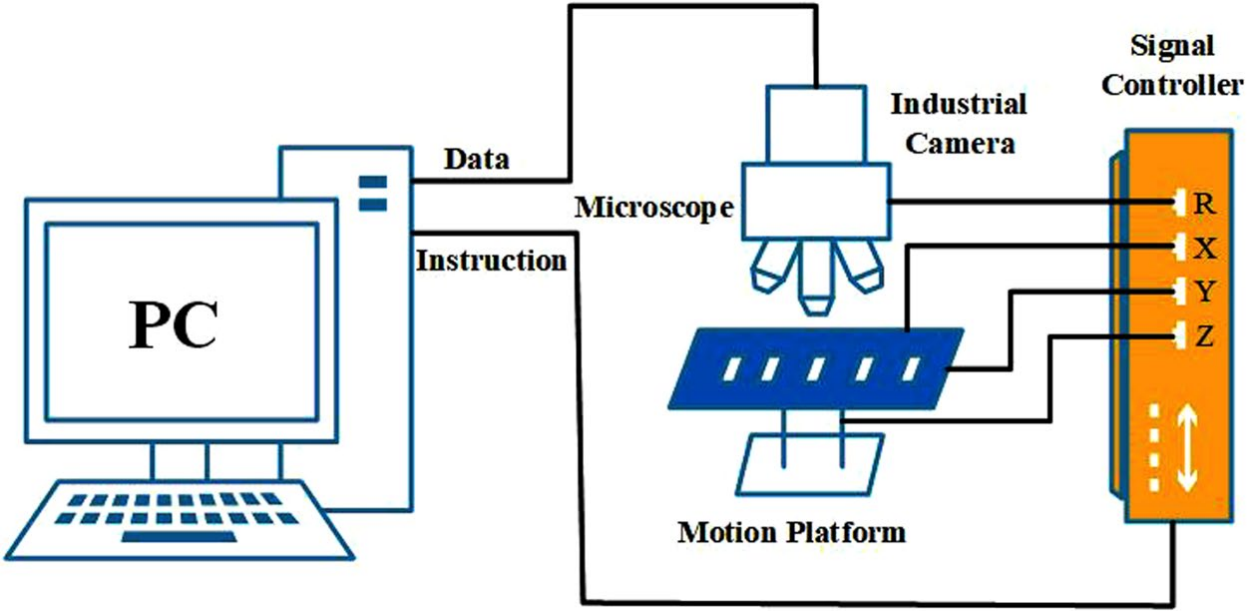
Dicentric chromosome identification system.

To perform the segmentation and identification of chromosome, a chromosome image of cells in metaphases must first be obtained. The chromosome images used in this article are derived from regular Giemsa-stained slides. The chromosome image acquisition system consists of three parts (Fig. 2): (1) Color microscope, using an OLYMPUS optical microscope with an oil-immersed 100X objective. (2) CCD camera, using Lumenera’s camera, the camera connected to the computer through the USB interface, real-time display and photographing. (3) Motion console and storage system. The computer sends instructions by Ethernet to make the motion console move according to the default path while the microscope moves up and down to photograph the captured chromosome clumps and store it in the computer.

The algorithms were developed in Visual Studio 2013, and the software was implemented by C++, which consists of four modules. They are image acquisition module, image processing module, image analysis module and data storage management analysis module. Software support library includes OpenCV and other third-party libraries. Std::thread in C++ 11 provides multi-threading parallel processing for dicentric chromosome analysis operations. Graphic Processing Unit(GPU) is used to accelerate the image processing.

### Chromosome segmentation

Segmentation is an important step in chromosome analysis. The segmentation of chromosome will affect chromosome centromere identification and the accuracy of karyotype analysis. The chromosome segmentation includes the segmentation of chromosomes and backgrounds, and the segmentation of chromosome clumps. And the segmentation of chromosome clumps is divided into simple adhesion segmentation and overlapped cross segmentation.

In this paper, the chromosomes and backgrounds are first segmented by clustering algorithm. The K-Means clustering algorithm is mainly used to implement the automatic clustering. It is an unsupervised machine learning algorithm and is widely used. There are many kinds of clustering algorithms. This paper uses the K-Means++ algorithm for segmentation. The main parameters of the K-Means++ algorithm are samples, clusterCount, termcrit, and attempts. The metaphase split phase images are taken as samples, and the clusterCount is chosen to be 2, which separates the background from the chromosome object, the termcrit is chosen to be 40, and the attempts is selected as 3. After the first clustering is completed, a large number of chromosome clusters are generated, and a small number of individual chromosomes are generated. By simple feature judgment, the screened chromosome clumps are re-segmented. For slightly clumped chromosome clumps, the watershed method is a commonly used segmentation method. In the paper, the clustering algorithm is combined with the watershed algorithm to segment the chromosome clumps. In the second segmentation, partial clustering algorithm parameters such as clusterCount and termcrit are appropriately adjusted for the chromosome clumps. After using the K-Means++ clustering algorithm, since the central parts of chromosomes are darkly stained at the opposite edges, this clustering algorithm makes the chromosomes thinner, that is, discarding the lighter part of the edge and retaining the deeper part of the center, thus making the large-scale adhesion chromosomes separate. However, the segmented chromosome is relatively thinner than the chromosome segmented in the first step. In order to ensure the consistency of the chromosome morphology of the whole cell, using the idea of watershed algorithm, the thinner segmented chromosome is used as a seed point. “Watering” one pixel at a time centered on each seed point until two different seed points meet the core “waters”. At this time, different chromosomes are labeled with the seed point as a reference point, and each chromosome can be separated according to the label.

**Figure 3:**
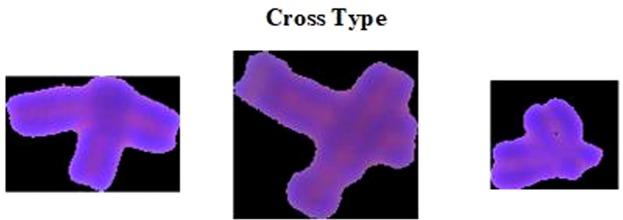
Examples of overlapping and cross chromosome clumps.

For overlapping chromosome clumps, although some papers propose some solution algorithms, such as threshold segmentation method^1^, deep learning after sampling from the artificially segmented chromosomes^2^, geometry-based segmentation method^3^, IAFCM (improved adaptive fuzzy C-Means algorithm)^5^, fuzzy c-means clustering algorithm and watershed algorithm^6^, CPOOS (classification-driven partially occluded object segmentation)^17^. But mostly for a specific type overlapping chromosomes, and segmentation does not have universal applicability, and even if the segmentation is performed according to the algorithms in the papers, the segmented single chromosomes are prone to misidentify the centromeres in the subsequent chromosome centromere identification algorithm. Therefore, for overlapping chromosome clumps (Fig. 3), this type is used to identify by manual interaction.

### Centerline extraction

Many operations on chromosomes require the centerline of chromosomes, such as classification of chromosomes^4,14^. Many features related to shape and structure, such as width and density profiles, can be extracted based on the centerline of the chromosome. The small deviations in extracting these real features may lead to identification and classification errors^18^. When the extraction of the chromosome centerline is completed, the identification and counting of the centromeres of single chromosomes can be performed according to the centerline.

This article’s processing method draws the minimum circumscribed rectangle of each chromosome firstly. Using the ratio of the area of the chromosome to the area of the smallest rectangle as a measure, when the ratio is less than a certain value, it indicates that the degree of chromosome bending is severe. When the ratio is close to 1, it indicates that the chromosome is straight. For the extended type chromosome, the axis of symmetry that parallels to the long side of the minimum circumscribed rectangle is directly used as the centerline of the chromosome. For the more severely curved chromosomes, use the method^19^ to extract the skeleton. In order to overcome the problem of small bifurcations and small holes when extracting the skeleton by this algorithm, the chromosomes are eroded, dilated, smoothed borders and filled the internal pores. To overcome the fact that the skeleton extracted by this algorithm is significantly shorter than the chromosome, the approximate slope is calculated at both ends of the skeleton, and then an empirical value length is extended to solve the extraction of short skeleton.

### Centromere identification

Centromeres are also called kinetochores. Chromosome centromeres refer to the pair of spherical structures that are located in the centromere area and the outer surface of the two chromatids and can be colored by special methods. The number of dicentric chromosomes in the human peripheral blood lymphocytes is used to detect the presence of chromosomal abnormalities in an individual. Or when a radiation accident occurs, the number of dicentric chromosomes is used to estimate the exposure of the human body to radiation. According to the radiation dose, it can improve the patient’s efficient and timely treatment.

Observing and analyzing the chromosome image, we find that there are three distinct differences in the image characteristics of the centromere and non-centromere area. The chromosome usually has a smaller width at the centromere, a smaller gray value, and the distribution of gray values is more uniform, so these differences are usually used as the characteristics of the centromere identification. For example, the projection vectors in the horizontal and vertical directions are calculated by adding up the values of the pixels along the projection line to determine the centromere position^12^. The identification of centromeres can be identified using fuzzy sets and neighborhood masks^13^. The pixel and distance are computed to find centromeres^14^. The identification of centromeres can also be used to calculate the number of centromeres by concavity^8^, but both angle and curvature can cause noise pollution^20^. The multiple identification methods of centromeres mainly use the width or gray values. The accuracy of these identification methods is not very high. Therefore, the method of combining width and gray values is used to identify the location of centromeres. The background gray value of the single chromosome after division is set to 0, and the gray value of the region of the chromosome itself is not processed. Let the coordinate of each point of the center axis extracted in II-B be *P*_*i*_(*x*_*i*_, *y*_*i*_), and the corresponding gray value is *M*_*i*_. Since the gray value is smaller, negate it, denote it as *G*_*i*_:

1 $${G}_{i}=255-{M}_{i}$$

At point *P*_*i*_, make a vertical line about the center axis. The coordinates of the point on each axis are ${P}_{i}{Q}_{j}({x}_{ij},\,{y}_{ij})$, the negation of the corresponding gray value is *G*_*ij*_, and the Euclidean distance from the vertical point *P*_*i*_ to the point *P*_*i*_*Q*_*i*_ in vertical line is *D*_*ij*_:

2 $${D}_{ij}=\sqrt{{({x}_{i}-{x}_{ij})}^{2}+{({y}_{i}-{y}_{ij})}^{2}}$$

Define a new parameter: the equivalent width *Y*_*i*_, then the equivalent width at point *P*_*i*_ is:

3 $${Y}_{i}=\sum {D}_{ij}^{m}\cdot {G}_{ij}^{n}(m,\,n\in N)$$

For a single chromosome after extracting the central axis, the process of identifying the centromere according to the equivalent width is as follows:

1. A single chromosome after extracting the central axis is taken as input, and the equivalent width curve of the point on the central axis is obtained, and one-dimensional low-pass filtering is performed thereon.
2. For the filtered equivalent width curve, the trend of the equivalent width is fitted with a straight line, and the difference between the point value on the fitted line and the value corresponding to the filtered point is used to generate a difference curve.
3. Derivate the difference curve and perform one-dimensional low-pass filtering to find all extreme points
4. For all the minimum values, find the difference between the maximum value of the left and right sides of the point, and the difference between the left side is recorded as A and the right side is recorded as B.
5. A threshold T is set according to a large amount of data. When A > T or B > T, and A > T/2 and B > T/2, the minimum point is a centromere point.

According to the number of centromere points, the chromosome can be judged as dicentric chromosomes, or monocentric chromosomes, or multicentric centromere chromosomes.

### Dose estimation

Radiation sources that are usually exposed to the human body are X rays, γ rays, and occasionally neutrons. The radiation dose estimation for humans are often using a γ ray curve. As the uncertainty of counting can be caused by slides or observation of individual differences in chromosome centromeres, a confidence interval is introduced to express uncertainty, using a 95% confidence interval as a criterion^21^. As the Poisson distribution of detected aberrations in the overexposed sample and the uncertainty in the calibration curve that is close to the normal distribution, it is difficult to calculate the confidence limits. Savage^22^, Merkle^23^, and Szluinska^24^ have been analyzed and discussed this problem. Merkle’s method is the simplest, and considers both the Poisson error on the aberration yield and the errors on the dose curve to be taken into account.

**Figure 4:**
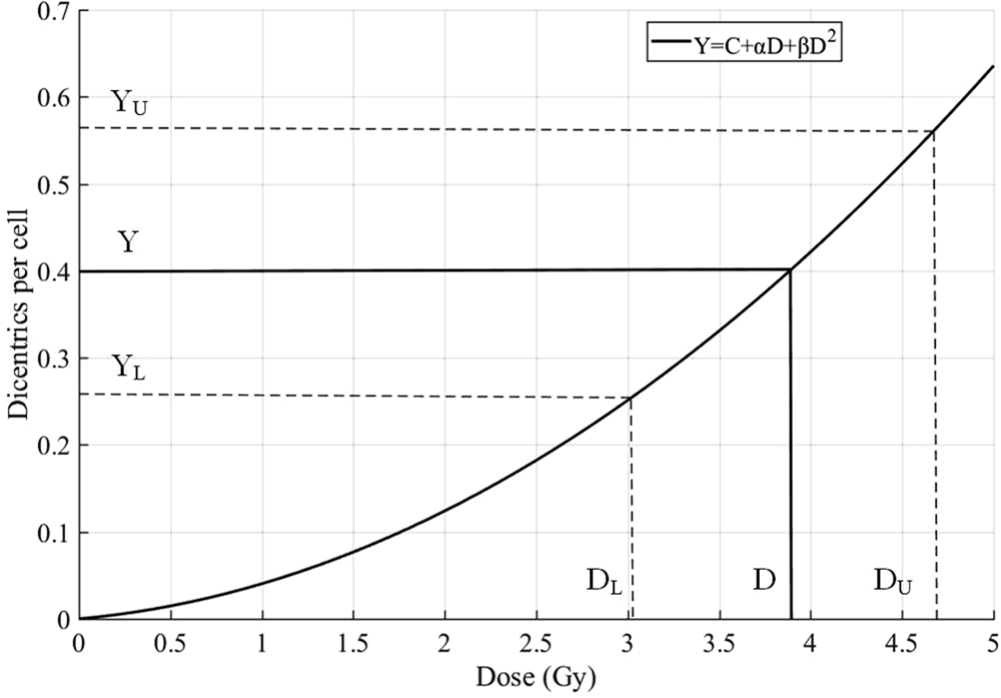
A dose-effect curve used to estimate uncertainties ignoring the error due to the curve.

For the dose-effect curve established on counting a large number of cells, the change of the curve is small compared with the change of the distortion rate of the subject, which can be neglected. As shown in the Fig. 4 and Table 1, the confidence interval can be calculated through the following four steps.

1. Assuming that M cells are analyzed and contain X dicentric chromosomes, the distortion yield is:
  4 $$Y=X/M$$
  The dose-effect curve is a linear square model(Y = C + αD + βD^2^), the estimated dose D can be obtained by solving the equation:
  5 $$D=\frac{-\,\alpha +\sqrt{{\alpha }^{2}+4\beta (Y-C)}}{2\beta }$$
2. Assuming the Poisson distribution, X_U_ and X_L_ are obtained from the standard statistical table of the expected Poisson’s distribution limit^25^. The Y_U_ and Y_L_ are:
  6 $${Y}_{U}={X}_{U}/M$$
  7 $${Y}_{L}={X}_{L}/M$$
3. Calculate the dose at the intersection of Y_L_ and the curve, which is the lower confidence limit(D_L_).
4. Calculate the dose at the intersection of Y_U_ and the curve, which is the upper confidence limit(D_U_).

**Table 1:** The Poisson upper and lower 95% confidence limits on observed numbers(X) of dicentrics^25^.

X	X_L_	X_U_, X_D_	X	X_L_	X_U_, X_D_	X	X_L_	X_U_, X_D_	X	X_L_	X_U_, X_D_
0	0	3.285	40	28.97	53.72	80	62.81	99.17	120	99.17	142.70
1	0.051	5.323	41	28.97	54.99	81	63.49	99.17	121	99.17	144.01
2	0.355	6.686	42	30.02	55.51	82	64.95	100.32	122	100.32	144.01
3	0.818	8.102	43	31.675	56.99	83	66.76	101.71	123	101.71	145.08
4	1.366	9.598	44	31.675	58.72	84	66.76	103.31	124	103.31	146.39
5	1.970	11.177	45	32.28	58.84	85	66.76	104.40	125	104.40	147.80
6	2.613	12.817	46	34.05	60.24	86	68.10	104.58	126	104.40	149.53
7	3.285	13.765	47	34.665	61.90	87	69.62	105.90	127	104.58	150.19
8	3.285	14.921	48	34.665	62.81	88	71.09	107.32	128	105.90	150.36
9	4.460	16.768	49	36.03	63.49	89	71.09	109.11	129	107.32	151.63
10	5.323	17.633	50	37.67	64.95	90	71.28	109.61	130	109.11	152.96
11	5.323	19.050	51	37.67	66.76	91	72.66	110.11	131	109.61	154.39
12	6.686	20.335	52	38.16	66.76	92	74.22	111.44	132	109.61	156.32
13	6.686	21.364	53	39.76	68.10	93	75.49	112.87	133	110.11	156.32
14	8.102	22.945	54	40.94	69.62	94	75.49	114.84	134	111.44	156.87
15	8.102	23.762	55	40.94	71.09	95	75.78	114.84	135	112.87	158.15
16	9.598	25.400	56	41.75	71.28	96	77.16	115.60	136	114.84	159.48
17	9.598	26.306	57	43.45	72.66	97	78.73	116.93	137	114.84	160.92
18	11.177	27.735	58	44.26	74.22	98	79.98	118.35	138	114.84	162.79
19	11.177	28.966	59	44.26	75.49	99	79.98	120.36	139	115.60	162.79
20	12.817	30.017	60	45.28	75.78	100	80.25	120.36	140	116.93	163.35
21	12.817	31.675	61	47.02	77.16	101	81.61	121.06	141	118.35	164.63
22	13.765	32.277	62	47.69	78.73	102	83.14	122.37	142	120.36	165.96
23	14.921	34.048	63	47.69	79.98	103	84.57	123.77	143	120.36	167.39
24	14.921	34.665	64	48.74	80.25	104	84.57	125.46	144	120.36	169.33
25	16.768	36.030	65	50.42	81.61	105	84.67	126.26	145	121.06	169.33
26	16.77	37.67	66	51.29	83.14	106	86.01	126.48	146	122.37	169.80
27	17.63	38.165	67	51.29	84.57	107	87.48	127.78	147	123.77	171.07
28	19.05	39.76	68	52.15	84.67	108	89.23	129.14	148	125.46	172.38
29	19.05	10.94	69	53.72	86.01	109	89.23	130.68	149	126.26	173.79
30	20.335	41.75	70	54.99	87.48	110	89.23	132.03	150	126.26	175.48
31	21.36	43.45	71	54.99	89.23	111	90.37	132.03	151	126.48	176.23
32	21.36	44.26	72	55.51	89.23	112	91.78	133.14	152	127.78	176.23
33	22.945	45.28	73	56.99	90.37	113	93.48	134.48	153	129.14	177.48
34	23.76	47.025	74	58.72	91.78	114	94.23	135.92	154	130.68	178.77
35	23.76	47.69	75	58.72	93.48	115	94.23	137.79	155	132.03	180.14
36	25.4	48.74	76	58.84	94.23	116	94.70	137.79	156	132.03	181.67
37	26.31	50.42	77	60.24	94.70	117	96.06	138.49	157	132.03	183.05
38	26.31	51.29	78	60.90	96.06	118	97.54	139.79	158	133.14	183.05
39	27.735	52.15	79	62.81	97.54	119	99.17	141.16	159	134.48	183.86

## Results and Discussion

### Segmentation results

The chromosome segmentation includes the segmentation of chromosomes and backgrounds, the segmentation of chromosome clumps, and the segmentation of chromosome clumps is divided into simple adhesion segmentation and overlapped cross segmentation.

**Figure 5:**
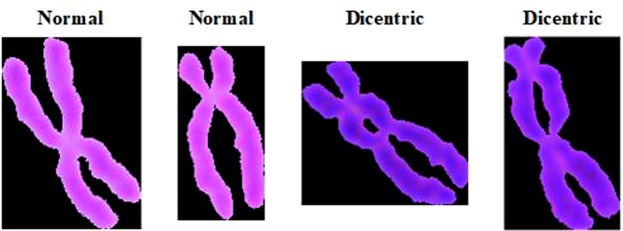
Examples of single chromosome.

After the chromosome and background segmentation of the original picture, the initial clustering segmentation can generate single chromosomes (Fig. 5). And when segmenting, the algorithm does not change the original morphology of the chromosome.

**Figure 6:**
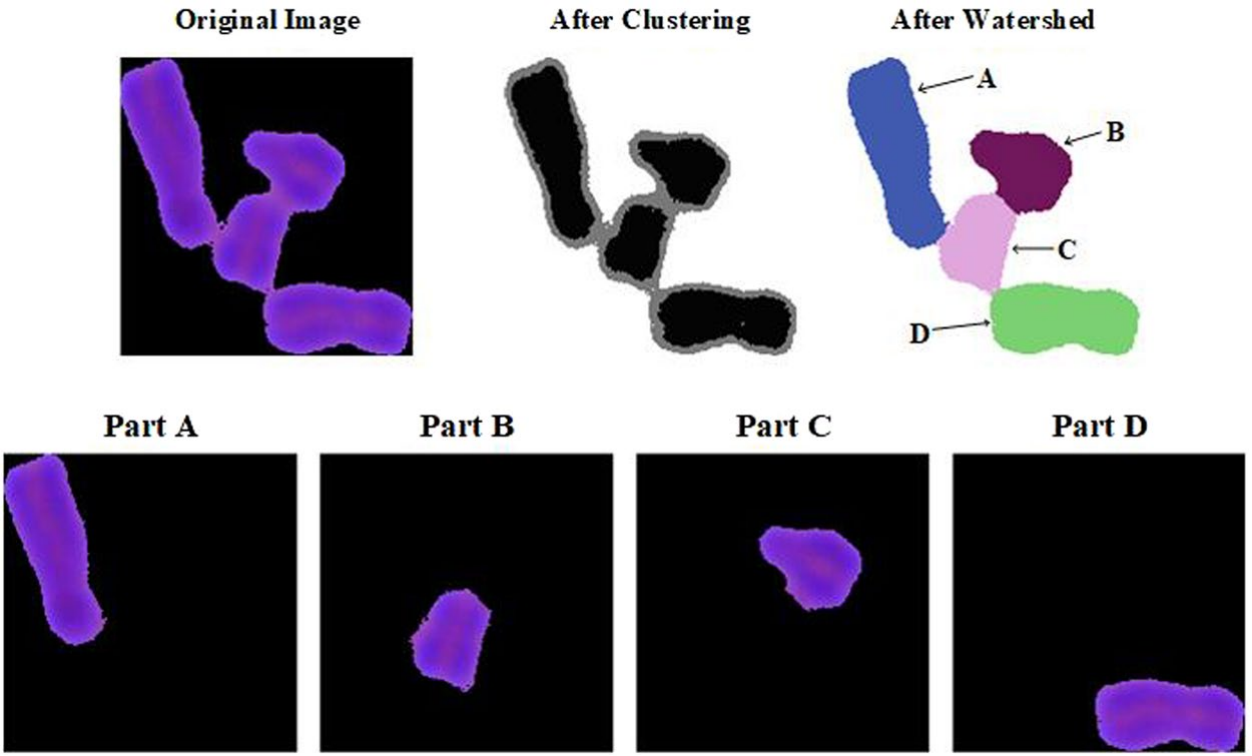
Segmentation process and result of final segmentation.

The simple characterization of the first-segmented chromosome clumps is performed, and the re-segmented chromosome clumps are selected. These selected chromosome clumps are taken as input to segment by clustering and watershed algorithm, it is obvious that the clustering segmented chromosomes are one circle smaller than the original chromosomes. In order to ensure the consistency of chromosome morphology, regarding the clustering segmented chromosomes as seed points, performing watershed segmentation can separate slightly sticky chromosome clumps (Fig. 6).

Most of the chromosomes are derived from human peripheral blood lymphocytes after gamma-irradiation. After being stained with Giemsa, they are placed on a microscope platform and scanned automatically. The data consist of metaphase split images taken from photographs of different doses of slides. Data contain 15,000 metaphase images, which are divided into data set 1, data set 2, and data set 3 for 5,000 images of 1 Gy,2 Gy,4 Gy radiation dose. And each data set contains 10 groups. For the data set 1, 2, 3, the software of using the clustering + watershed algorithm segmented the average group of 19542, 20128, 20732 objects, averaging 39,40,41 objects per metaphase. When the threshold is used, the software segmented the average group of 14654, 15244, 16178 objects, averaging 29, 30, 32 objects per metaphase.

### Extraction centerline results

**Figure 7:**
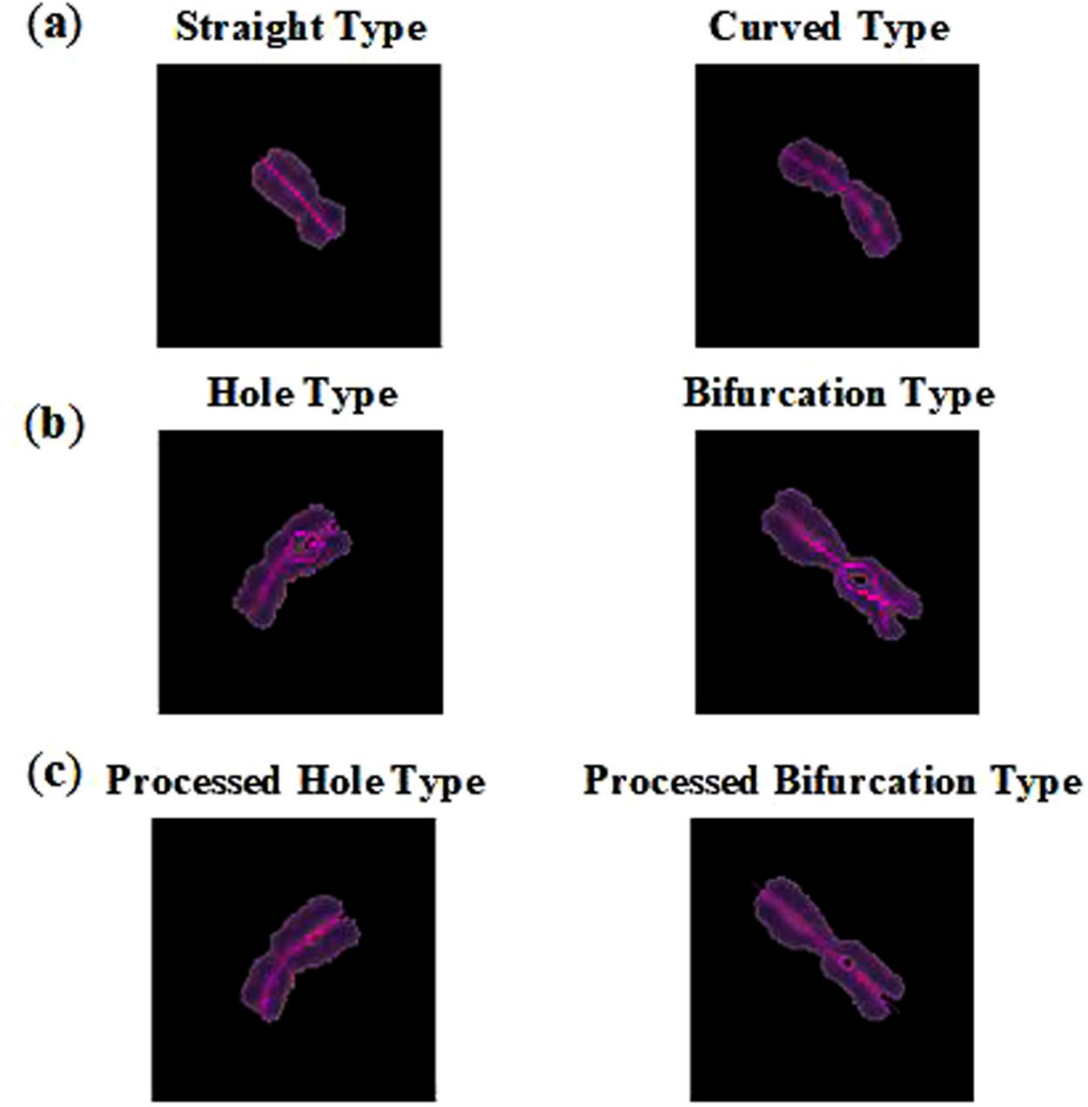
Extraction centerline results of four type chromosomes.

The centerline is extracted from the single segmented chromosomes. For the straight type, or curved type can directly obtain the centerline (Fig. 7a). For the hole type, or bifurcation type, the centerline can be obtained after the chromosome has been eroded, dilated, smoothed borders and filled the internal pores (Fig. 7b,c). The centerlines of most single chromosomes can be extracted, except for some specially shaped chromosomes. The centerlines of these chromosomes will produce a severe shift. However, since the occurrences of this type is infrequent, the effect on the dicentric chromosome recognition results almost can be neglect.

### Dicentric chromosome identification results

**Figure 8:**
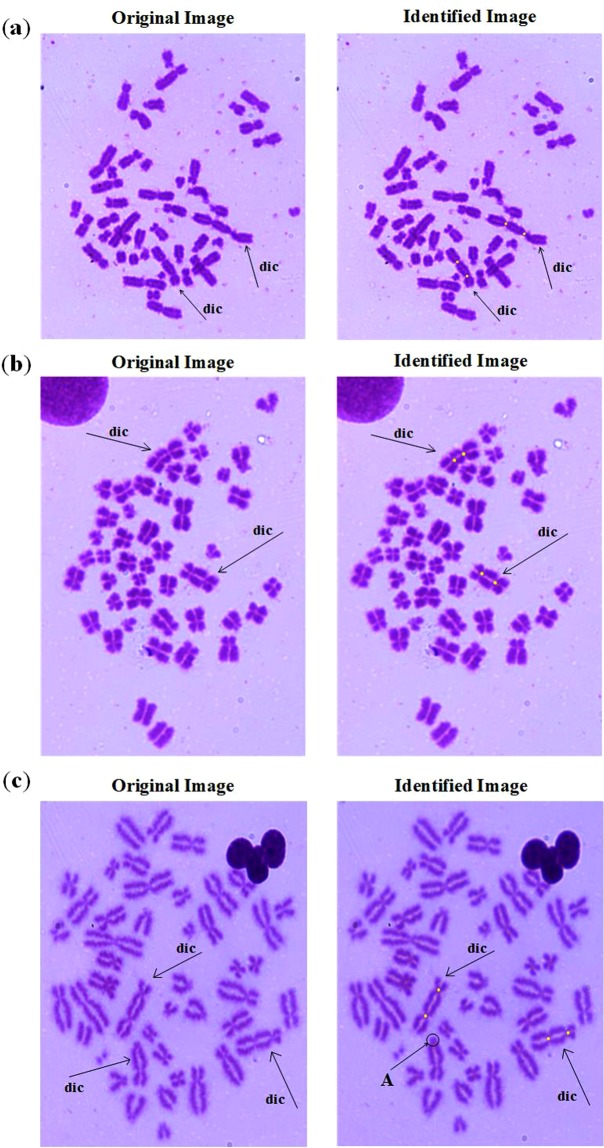
Identification results of dicentric chromosomes in single cells.

As shown in II-A, a new method for chromosome segmentation is designed based on the clustering algorithm and the watershed algorithm. The segmented single chromosome can be identified centromeres according to the algorithm in II-B and II-C. The identification results of dicentric chromosomes are showed in Fig. 8. It can be clearly seen that Fig. 8a,b have two dicentric chromosomes which are identified correctly. But Fig. 8c contains three dicentric chromosomes, only two dicentric chromosomes have been identified because there is no outwardly extending chromosome arm at the unrecognized part A. Therefore, it is more difficult to identify this type dicentric chromosome.

Foe three data sets, experts identified all dicentric chromosome and also labeled false positive dicentric chromosomes. The experts also judged the identified dicentric chromosomes after the software identification and corrected the number of identified dicentric chromosomes.

**Figure 9:**
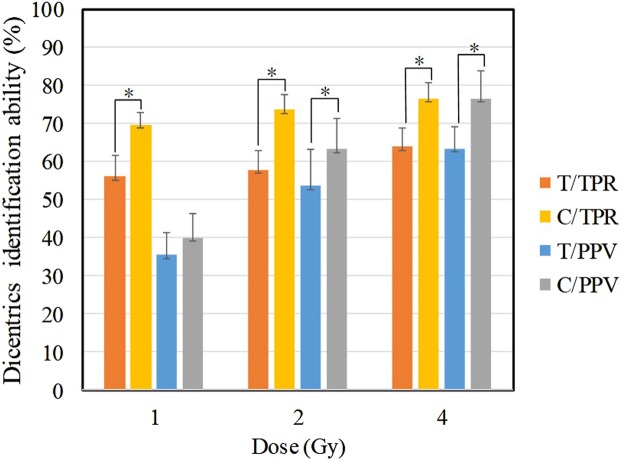
Effects of clustering + watershed algorithm and threshold algorithm on dicentric chromosome identification. Orange signals indicate the true positive rate by threshold algorithm. Yellow signals indicate the true positive rate by clustering + watershed algorithm. Blue signals indicate the positive predictive value by threshold algorithm. Gray signals indicate the positive predictive value by clustering + watershed algorithm. Mean ± S.D., n = 10, *P < 0.05.

The dicentric chromosomes in three data sets were identified for the (m = 1, n = 1) values and compared with the threshold algorithm, the results are shown in Fig. 9. PPV and TPR are used to measure the identification of algorithms among different methods. PPV indicates the identification accuracy of the dicentric chromosomes, and TPR indicates the identification rate of the dicentric chromosomes. As can be seen from Fig. 9, compared with the threshold algorithm, the clustering + watershed algorithm has better results on TPR and PPV. Especially in high-dose radiation, the clustering + watershed algorithm has the TPR of 76.6% and the PPV of 76.6%, both of which exceed three-quarters, showing good identification results. At low dose, due to the relatively low radiation dose, the formation of dicentric chromosomes is less, normal chromosomes are more. It is prone to mis-segmentation, which will lead to low identification accuracy (30–40%).

### Dose estimation results

**Figure 10:**
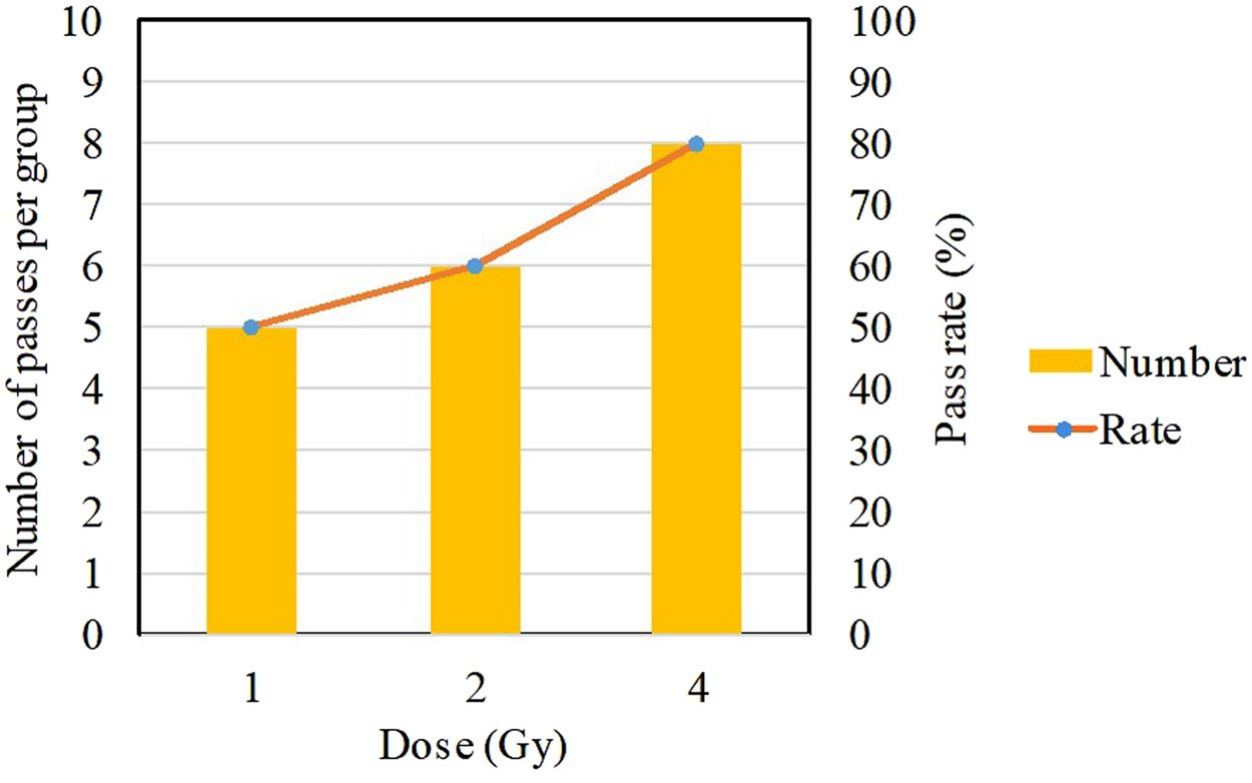
Results of dose estimation for three data sets.

Dose estimation was performed on 30 groups of identification results. The dose curve was based on the dose-effect curve of the dicentric chromosomes fitted in our laboratory (8). The relative deviation of the estimated dose ≤20% is regarded as qualified. The pass rate for each data set is shown in Fig. 10.

8 $$Y=0.000105+0.0186\,D+0.0217\,{D}^{2}$$

As can be seen from Fig. 10, the higher dose, the higher pass rate of the dose estimate. Therefore, when dose estimation is performed, the number of cells should be selected more for low-dose radiation, and the number of cells can be appropriately reduced for high doses.

## Conclusion

This paper proposes a segmentation method based on clustering algorithm and watershed algorithm to segment the chromosome cluster, and then extract the central axis from the segmented single chromosome. According to the position of the central axis, the dicentric chromosomes are identified by combining the two factors of gray scale and distance. After manually identifying the identified dicentric chromosomes, the number of dicentric chromosomes is obtained, which can be used to estimate radiation doses. The results are as follows:

1. The proposed automatic segmentation and identification method for dicentric chromosomes has the true positive rate (TPR) 75.6% and the positive predictive value (PPV) of 60%, which is higher than the method using threshold algorithm.
2. By comparing the different dose, it is found that the higher dose, the higher true positive rate and positive predictive value can be obtained, especially the positive predictive value.
3. The yield and pass rate of dose estimation depend on the amount of radiation dose received. For low dose radiation, the more cells should be identified, and for high dose radiation, the number of identified cells can be appropriately reduced. When 500 cells are used for identification and dose estimation, the dose estimation pass rate can reach 80% in high dose.

### Statistics

Data were tested for normal distribution. Differences between groups were analyzed using the paired Student t test (IBM SPSS Statistics v. 17.0, IBM, Armonk, NY). All values are expressed as mean ± standard deviation (SD). Statistical significance was accepted for values of P < 0.05.

## Data Availability

The datasets generated and/or analyzed during the current study are available from the corresponding author on reasonable request.
